# 6-gingerol modulates lipid metabolism, insulin resistance, and inflammation in NAFLD: evidence from a preclinical meta-analysis

**DOI:** 10.3389/fnut.2026.1872764

**Published:** 2026-08-13

**Authors:** Dachuan Jin, Shunqin Jin, Tao Zhou, Hongzhi Li, Guangming Li, Chuan Qin, Wubing Ying, Guoping Sheng, Peng Gao

**Affiliations:** 1 Department of Tuberculosis, Hepatology, and Pharmacy, Translational Medicine Research Center, Zhengzhou Sixth People’s Hospital, Zhengzhou, China; 2 Department of Nuclear Medicine, The First Medical Center, Chinese PLA General Hospital, Beijing, China; 3 Department of Geriatric Medicine and Laboratory of Gerontology and Anti-Aging Research, Qilu Hospital of Shandong University, Jinan, China; 4 Department of Infectious Diseases, Key Laboratory of Artificial Organs and Computational Medicine of Zhejiang Province, Shulan (Hangzhou) Hospital, Shulan International Medical College, Zhejiang Shuren University, Hangzhou, China

**Keywords:** 6-gingerol, insulin resistance, lipid metabolism, non-alcoholic fatty liver disease, preclinical meta-analysis

## Abstract

**Background:**

Non-alcoholic fatty liver disease (NAFLD) is a major global health concern with limited effective pharmacological treatments. 6-Gingerol, a principal bioactive compound of ginger, has shown metabolic and hepatoprotective potential in experimental studies; however, its overall efficacy has not been systematically quantified.

**Objective:**

To evaluate the effects of 6-gingerol on NAFLD-related outcomes in animal models and to explore potential sources of heterogeneity.

**Methods:**

A systematic review and meta-analysis of controlled animal studies was conducted in accordance with PRISMA guidelines. Multiple databases were searched to identify relevant studies. Standardized mean differences (SMDs) with 95% confidence intervals (CIs) were calculated using random-effects models. Prespecified subgroup analyses were performed based on species, dose, and treatment duration, and sensitivity analyses assessed the robustness of the results.

**Results:**

Eight studies were included. 6-Gingerol significantly improved anthropometric indices, hepatic and circulating lipid profiles, liver enzymes, and glucose metabolism parameters. Beneficial effects were also observed for inflammatory mediators and adipokines in a subset of studies. Despite moderate to high heterogeneity, favorable effects were observed across the evaluated major outcome domains. Exploratory subgroup analyses suggested that treatment duration, dose, and species may contribute to between-study variability; however, the small number of studies within several subgroup strata precluded firm conclusions regarding effect modification. Sensitivity analyses showed that the overall direction of the pooled estimates was not materially changed by removal of individual studies.

**Conclusion:**

6-Gingerol was associated with favorable changes in several metabolic and hepatic outcomes in animal models of NAFLD. However, these findings should be interpreted cautiously because of the limited number of studies, substantial heterogeneity, and incomplete methodological reporting. Further standardized preclinical and clinical studies are warranted.

**Systematic review registration:**

https://www.crd.york.ac.uk/PROSPERO/view/CRD420261290223, identifier CRD420261290223.

## 1 Introduction

6-Gingerol, the principal bioactive phenolic compound derived from Zingiber officinale (ginger), is widely regarded as a primary mediator of the pharmacological properties attributed to ginger (1–3). This natural product exhibits a spectrum of pleiotropic biological actions, including well-documented antioxidant and anti-inflammatory effects, as well as modulatory influences on metabolic homeostasis (4, 5). These multifaceted properties have supported interest in 6-gingerol as a potential candidate for addressing metabolic disorders characterized by lipid dysregulation, oxidative stress, and chronic low-grade inflammation.

Non-alcoholic fatty liver disease (NAFLD) has emerged as a predominant cause of chronic liver pathology globally, closely associated with obesity, insulin resistance, and heightened cardiometabolic risk (6–8). Its pathogenesis is a complex, multi-hit process involving the interplay of hepatic steatosis, impaired insulin signaling, oxidative damage, and pro-inflammatory cascades (9, 10). These interconnected mechanisms collectively drive the transition from simple steatosis to more severe non-alcoholic steatohepatitis (NASH) and fibrosis. Despite extensive research, effective pharmacological interventions for NAFLD remain scarce, fueling continued interest in bioactive natural products that can simultaneously target multiple pathogenic pathways with favorable safety profiles (8, 11).

In recent years, a substantial body of preclinical research has evaluated the efficacy of 6-gingerol in various dietary or genetically induced animal models of NAFLD (4, 12–15). While many of these controlled studies report improvements in histopathological features and metabolic parameters, the findings are heterogeneous, varying considerably with experimental models, species, dosing regimens, and treatment durations. Consequently, the consistency and magnitude of these therapeutic effects have yet to be systematically quantified. Furthermore, the reported mechanistic insights are often fragmented and inconsistent, impeding a cohesive understanding of how 6-gingerol modulates disease-relevant pathways *in vivo* and hindering the translation of these findings.

To address this gap, we conducted a systematic review and meta-analysis of controlled animal intervention studies. Our objective was to quantitatively evaluate the efficacy of 6-gingerol in preclinical NAFLD models across key outcome domains, including hepatic steatosis, lipid profiles, liver injury biomarkers, and indices of glucose/insulin resistance. Additionally, we aimed to narratively synthesize the mechanistic signals reported in the included studies to construct a more integrated picture of its action. By consolidating and critically appraising the existing preclinical evidence, this work seeks to clarify the overall therapeutic potential of 6-gingerol, identify critical sources of experimental heterogeneity, and provide an evidence-based summary to guide future standardized preclinical investigations and inform the design of translational research.

## 2 Materials and methods

### 2.1 Protocol registration and reporting standards

This systematic review and meta-analysis was conducted in accordance with the Preferred Reporting Items for Systematic Reviews and Meta-Analyses (PRISMA) 2020 statement (16). The review protocol was prospectively registered in the International Prospective Register of Systematic Reviews (PROSPERO) under the registration number CRD420261290223. A PRISMA 2020 flow diagram was used to document the study selection process.

### 2.2 Search strategy

We conducted a comprehensive literature search to identify controlled animal intervention studies evaluating the effects of 6-gingerol in experimental models of NAFLD. PubMed/MEDLINE, Embase, Web of Science Core Collection, and the Cochrane Central Register of Controlled Trials (CENTRAL) were searched from inception to 21 January 2026. A detailed PubMed search strategy is provided in Table 1 as a representative example, whereas the complete search strategies for the remaining databases are provided in Supplementary Tables 1–3.

**TABLE 1:** Search strategy.

Search ID	Search query
#1	Gingerol OR (6)-gingerol OR 6-gingerol
#2	Non-alcoholic Fatty Liver Disease
#3	Non-alcoholic Fatty Liver Disease OR Non-alcoholic Fatty Liver Disease OR Fatty Liver, Nonalcoholic OR Fatty Livers, Nonalcoholic OR Liver, Nonalcoholic Fatty OR Livers, Nonalcoholic Fatty OR Nonalcoholic Fatty Liver OR Nonalcoholic Fatty Livers OR NAFLD OR Nonalcoholic Fatty Liver Disease OR Nonalcoholic Steatohepatitis OR Nonalcoholic Steatohepatitides OR Steatohepatitides, Nonalcoholic OR Steatohepatitis, Nonalcoholic OR MAFLD OR metabolic associated fatty liver disease OR MASLD OR metabolic dysfunction-associated steatotic liver disease OR metabolic dysfunction-associated fatty liver disease OR NAFLD OR NASH OR MASH OR metabolic associated steatohepatitis OR steatosis of liver OR steatohepatitis nonalcoholic OR metabolic associated steatohepatitis OR liver steatosis
#4	#2 OR #3
#5	#1 AND #4

No restrictions on language or publication date were applied. Reference lists of included studies and relevant reviews were manually screened to identify additional eligible records.

### 2.3 Eligibility criteria

### 2.3.1 Study design

In this review, NAFLD was used as an umbrella term encompassing both NAFLD and NASH experimental models. Controlled *in vivo* animal intervention studies were eligible for inclusion.

#### 2.3.2 Animal models

Eligible studies employed *in vivo* animal models of NAFLD or NASH, including diet-induced models (e.g., high-fat diet, high-fat/high-fructose diet, fructose-enriched diet, or methionine–choline-deficient diet). Any species or strain, either sex, and any age were eligible, provided that NAFLD/NASH was established and supported by histological and/or biochemical evidence.

#### 2.3.3 Interventions and comparators

The intervention of interest was 6-gingerol administered as monotherapy *in vivo*, regardless of dose, formulation, route of administration (e.g., oral gavage, diet or drinking water admixture, or injection), dosing frequency, or timing relative to NAFLD induction (preventive or therapeutic designs).

Eligible comparators included NAFLD model animals receiving vehicle/placebo, untreated NAFLD controls, or identical diet/drinking water without 6-gingerol. Studies were excluded if 6-gingerol was co-administered with other active compounds and its independent effect could not be isolated, or if ginger extracts or mixtures without clear identification and quantification of 6-gingerol were used.

#### 2.3.4 Outcomes

Studies were eligible if they reported at least one NAFLD-relevant outcome with extractable quantitative data for both intervention and control groups. Eligible outcomes included anthropometric indices, lipid metabolism measures (hepatic and/or serum), liver injury enzymes, glucose metabolism and insulin resistance parameters, oxidative stress markers, inflammatory mediators, and signaling molecules related to lipid metabolism, inflammation, or oxidative stress pathways.

### 2.4 Study selection

All retrieved records were imported into reference management software and duplicates were removed prior to screening. Two reviewers independently screened titles and abstracts against predefined inclusion and exclusion criteria, followed by full-text assessment of potentially eligible studies. Reasons for exclusion at the full-text stage were recorded. Disagreements were resolved through discussion, with adjudication by a third reviewer when necessary. The study selection process is summarized using a PRISMA 2020 flow diagram.

### 2.5 Data extraction

Data extraction was performed independently by two reviewers using a piloted, standardized extraction form. Extracted information included study characteristics, animal and model details, intervention and comparator characteristics, and quantitative outcome data (group-level means, measures of dispersion, and sample sizes).

When outcome data were presented only in graphical form, values were independently extracted from the figures by two reviewers using digital plot digitization software, and any discrepancies were resolved through discussion. Where required, standard formulas were used to convert standard errors of the mean (SEMs) or confidence intervals to standard deviations. When a study included multiple 6-gingerol dose arms sharing a common control group, only one intervention arm was retained for the primary quantitative synthesis to avoid double-counting the control group and violating the independence assumption. In accordance with the prespecified protocol, the highest-dose arm was selected a priori on the basis of dose level and without reference to the observed results, thereby providing a consistent arm-selection rule across studies. The potential influence of this arm-selection strategy was further examined in a sensitivity analysis, as described in section 2.7 (11).

### 2.6 Risk of bias assessment

Risk of bias in included studies was independently assessed by two reviewers using Systematic Review Centre for Laboratory Animal Experimentation (SYRCLE) Risk of Bias tool for animal studies (17). Each domain was judged as low, high, or unclear risk of bias based on reported methodological details. Discrepancies were resolved by consensus or third-reviewer adjudication. Risk-of-bias assessments were considered when interpreting pooled results.

### 2.7 Statistical analysis

All meta-analyses were conducted using random-effects models to account for anticipated between-study heterogeneity inherent to preclinical animal experiments, including differences in species, NAFLD induction methods, dosing regimens, and outcome assessment. For continuous outcomes, effect sizes were expressed as standardized mean differences (SMDs) with 95% confidence intervals. SMDs were selected because the included studies differed in animal species and strains, baseline outcome ranges, laboratory assessment methods, and, for some endpoints, reporting units or normalization procedures. Consequently, absolute mean differences could not be assumed to be consistently comparable across all studies, even when some outcomes were reported in nominally similar units. The use of SMDs therefore allowed treatment effects to be expressed on a common dimensionless scale across heterogeneous preclinical experiments.

Statistical heterogeneity was assessed using Cochran’s Q test and quantified with the I^2^ statistic. Only outcomes reported by three or more independent studies were quantitatively synthesized. Oxidative stress-related outcomes were evaluated in several studies, but different biomarkers were assessed and no individual marker was reported by at least three independent studies. Consequently, these outcomes were not quantitatively pooled.

Prespecified subgroup analyses were conducted to explore potential sources of heterogeneity according to animal species, 6-gingerol dose, and treatment duration, when data were sufficient to allow stratified analyses. Given the limited number of eligible preclinical studies, some subgroup strata were based on single studies; these analyses were considered exploratory and interpreted with caution. Meta-regression was not performed because fewer than 10 independent studies contributed to each outcome. Under these conditions, meta-regression would have limited statistical power and could produce unstable or spurious associations. We therefore restricted the exploration of heterogeneity to the prespecified subgroup analyses.

Sensitivity analyses were conducted for outcomes with at least five studies to assess the robustness of pooled estimates (18, 19). Outcomes with fewer studies were not subjected to sensitivity analyses because of limited statistical stability. In addition, to assess the robustness of the primary arm-selection strategy, a sensitivity analysis was conducted in which the highest-dose arm was replaced with the lowest-dose arm in studies reporting multiple 6-gingerol doses, while studies with only a single treatment dose were retained unchanged. Statistical significance was defined as a two-sided *P* < 0.05.

## 3 Results

### 3.1 Study selection

**FIGURE 1:**
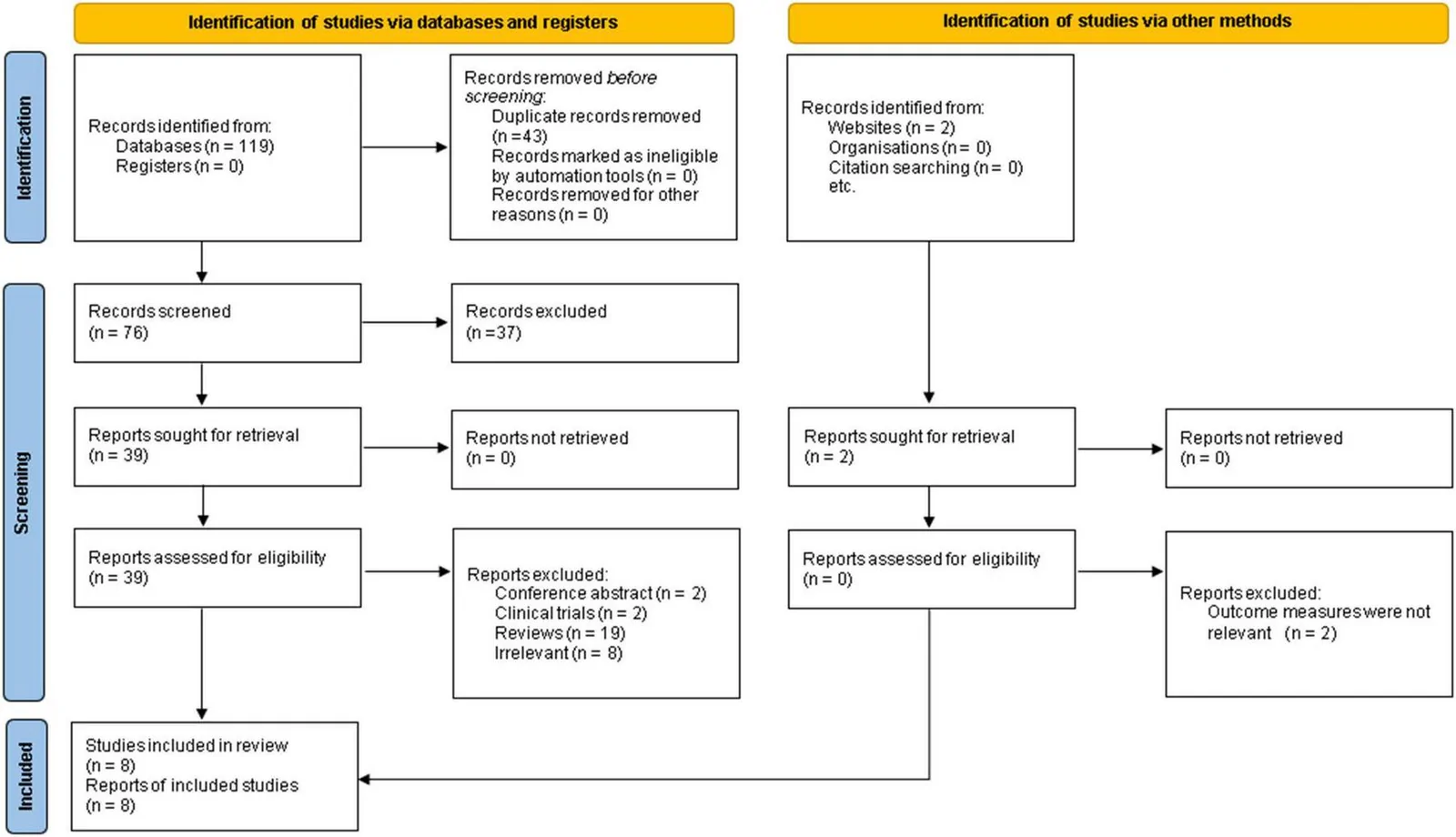
PRISMA 2020 flow diagram illustrating the study selection process. A total of 121 records were identified through database searching and other sources. After removal of duplicates, titles and abstracts were screened, followed by full-text assessment. Eight studies met the predefined inclusion criteria and were included in the qualitative synthesis and meta-analysis.

The literature search yielded 121 records (119 from electronic databases and two from other sources). After removal of 43 duplicates, 76 records were screened by title and abstract. Thirty-seven records were excluded at this stage. Full texts of 39 reports were assessed for eligibility, of which 31 were excluded for the following reasons: conference abstracts (*n* = 2), clinical studies (*n* = 2), review articles (*n* = 19), irrelevant content (*n* = 8), and outcomes not relevant to the present review (*n* = 2). No records were excluded due to retrieval failure. Ultimately, eight studies met the predefined inclusion criteria and were included in the qualitative synthesis and meta-analysis (4, 12–15, 20–22). The study selection process is summarized in the PRISMA 2020 flow diagram (Figure 1).

### 3.2 Study characteristics

Of the eight included studies, four were conducted in China (4, 15, 20, 22), two in Korea (13, 14), one in Indonesia (21), and one in Iran (12); all used rodent models of NAFLD/NASH, including rats (4, 21), mice (4, 12–15, 20), and Golden Syrian hamsters (22). In total, 138 animals were included across the eight studies. Across all experiments, 6-gingerol was administered as monotherapy and compared with appropriate NAFLD/NASH model controls (vehicle/placebo, untreated model controls, or identical diet/drinking water without 6-gingerol). Key study and intervention characteristics are summarized in Table 2.

**TABLE 2:** Characteristics of included studies.

Author-year	Country	Species (strain)	Age (w)	sex	n(I/C)	Model induction method	Dose	D (w)	ROA	Outcomes of interest
Ahn et al. (13)	Korea	Mice (C57BL/6J)	6	m	10/10	High-fat diet	NA	8	Dietary	Liver weight, hepatic TG/TC
Hong et al. (14)	Korea	Mice (C57BL/6J)	4	m	10/10	High-fat diet	NA	8	Dietary	BW, blood TG/TC/HDL-C/FFA, FBG, insulin, HOMA-IR, adiponectin, leptin
Li et al. (4)	China	Rat (SD)	5–6	m	8/8	High-fructose diet	2 mg/kg/d	4	Gavage	BW, liver index, blood TG/TC, hepatic TG/TC
Li et al. (4)	China	Mice (C57BL/6J, SCD1 +/+)	8–10	m	6/6	High-fructose diet	2 mg/kg/d	2	Gavage	BW, liver index, hepatic TG/TC
Liu et al. (20)	China	Mice (C57BL/6J)	6	m	6/6	High-fat diet	10/20 mg/kg/d	5	Gavage	BW, liver weight, liver index, blood TG/TC, hepatic TG/TC, FBG, insulin, HOMA-IR, ALT, AST, TNF-α, IL-6, FAS
Gunawan et al. (21)	Indonesia	Rat (SD)	8	m	5/5	Hfhf	50/100/200 mg/kg/d	8	Gavage	BW, liver weight, liver index, blood TG/TC/HDL-C/LDL-C, ALT, AST
Sarrafan et al. (12)	Iran	Mice (C57BL/6)	NA	m	6/6	High-fat diet	400/800 mg/kg/d	4	Gavage	BW, liver weight, liver index, blood HDL-C, LDL-C, hepatic TG/TC, FBG, insulin, HOMA-IR, ALT, AST, adiponectin, leptin
Xia et al. (15)	China	Mice (C57BL/6)	6	m	10/10	High-fat diet	100 mg/kg/d	4	Gavage	BW, liver weight, liver index, blood TG/TC/HDL-C/LDL-C/FFA, insulin, HOMA-IR, ALT, AST, TNF-α, IL-6, FAS
Tzeng et al. (22)	China	Golden Syrian hamster	8	m	8/8	HFD	25/50/100 mg/kg/d	8	Gavage	BW, liver index, blood TG/TC/HDL-C/LDL-C/FFA, hepatic TG/TC, FBG, insulin, TNF-α, IL-6, FAS, adiponectin, leptin

w, weeks; m, male; I/C, intervention/control; D, duration; ROA, route of administration; TG, triglycerides; TC, total cholesterol; HDL-C, high-density lipoprotein cholesterol; LDL-C, low-density lipoprotein cholesterol; FFA, free fatty acid; FAS, fatty acid synthase; FBG, fasting blood glucose; HOMA-IR, homeostatic model assessment for insulin resistance; BW, body weight; ALT, alanine aminotransferase; AST, aspartate aminotransferase; TNF, tumor necrosis factor; IL-6, interleukin-6.

### 3.3 Risk of bias assessment

Risk of bias was assessed using SYRCLE Risk of Bias tool, and the results are presented in Supplementary Figures 1, 2. Overall, the included studies were characterized by low or unclear risk of bias, with no domain judged as high risk. Most concerns arose from insufficient reporting of methodological details rather than evidence of definite methodological flaws. Incomplete outcome data and other bias were consistently judged as low risk across all included studies. Selective reporting was also assessed as low risk in the included studies, as no apparent discrepancies between the reported outcomes and study objectives were identified. In contrast, allocation concealment, blinding (detection bias), and random outcome assessment were generally rated as unclear risk in most studies due to limited reporting. Random sequence generation, baseline characteristics, blinding (performance bias) and random housing showed mixed judgments, with a proportion of studies reporting adequate methods and others remaining unclear. Taken together, the internal validity of the included studies was primarily limited by insufficient reporting of key methodological procedures.

### 3.4 Overall effects of 6-gingerol in NAFLD animal models

Across included studies, quantitative syntheses suggested that 6-gingerol improved multiple NAFLD-relevant outcome domains in experimental animals. Compared with NAFLD/NASH controls, 6-gingerol was associated with reductions in anthropometric indices (body weight, liver weight, and liver index), improvements in hepatic and circulating lipid profiles, lower liver injury enzymes [alanine aminotransferase (ALT) and aspartate aminotransferase (AST)], and favorable changes in glucose metabolism and insulin resistance markers [fasting blood glucose (FBG), insulin, and homeostatic model assessment of insulin resistance (HOMA-IR)]. Mechanistic endpoints further supported reductions in pro-inflammatory mediators and lipogenic signaling, alongside favorable modulation of adipokines. Substantial between-study heterogeneity was frequently observed, likely reflecting differences in species/strain, NAFLD induction methods, dosing regimens, and treatment duration. Prespecified subgroup and sensitivity analyses were therefore used to explore heterogeneity and test robustness.

### 3.5 Anthropometric outcomes

**FIGURE 2:**
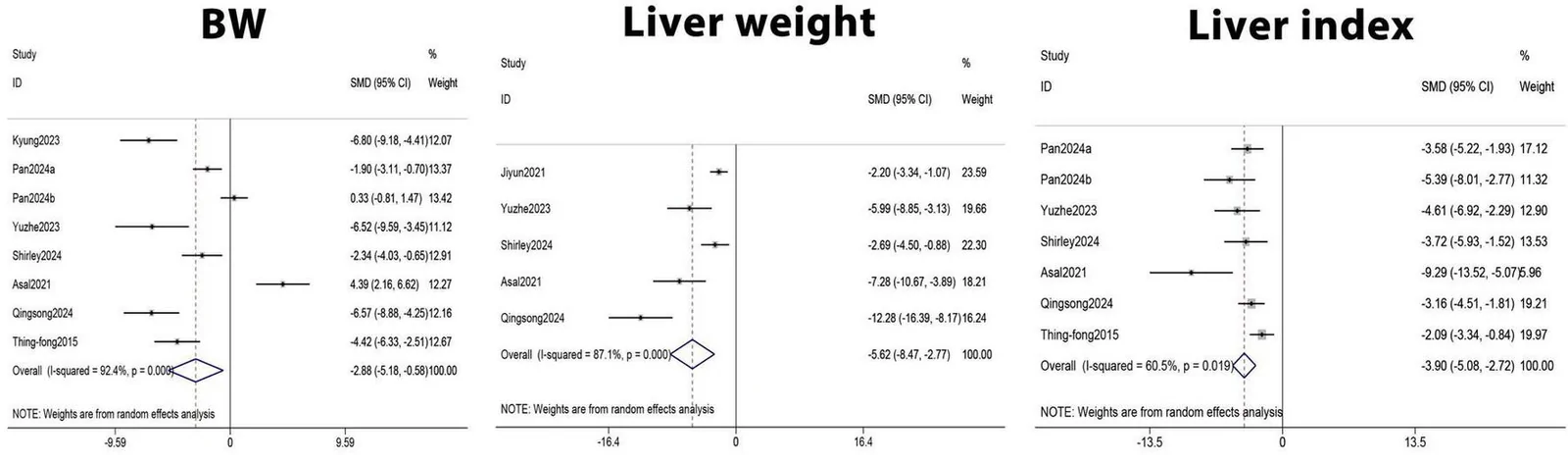
Forest plots showing the effects of 6-gingerol on anthropometric outcomes in experimental NAFLD animal models. Effect sizes are presented as SMDs with 95% CIs using random-effects models. Heterogeneity was assessed using the I^2^ statistic. Negative SMD values indicate favorable effects of 6-gingerol for body weight, liver weight, and liver index. NAFLD, non-alcoholic fatty liver disease; BW, body weight; CI, confidence interval; SMD, standardized mean difference.

Meta-analyses showed that 6-gingerol significantly improved anthropometric measures in NAFLD animal models (Figure 2). Compared with NAFLD controls, 6-gingerol reduced body weight (8 studies; SMD -2.88, 95% CI −5.18 to −0.58; *P* = 0.014), with substantial heterogeneity (*I*^2^ = 92.5%, *P* < 0.001). Liver weight was also reduced (5 studies; SMD −5.62, 95% CI −8.47 to −2.77; *P* < 0.001; *I*^2^ = 87.1%, *P* < 0.001). The liver index was significantly lower in treated animals (7 studies; SMD −3.90, 95% CI −5.08 to −2.72; *P* < 0.001), with moderate heterogeneity (*I*^2^ = 60.5%, *P* = 0.019). Overall, these findings indicate significant reductions in body and liver mass−related indices, although effect magnitudes varied across studies.

### 3.6 Lipid profiles

**FIGURE 3:**
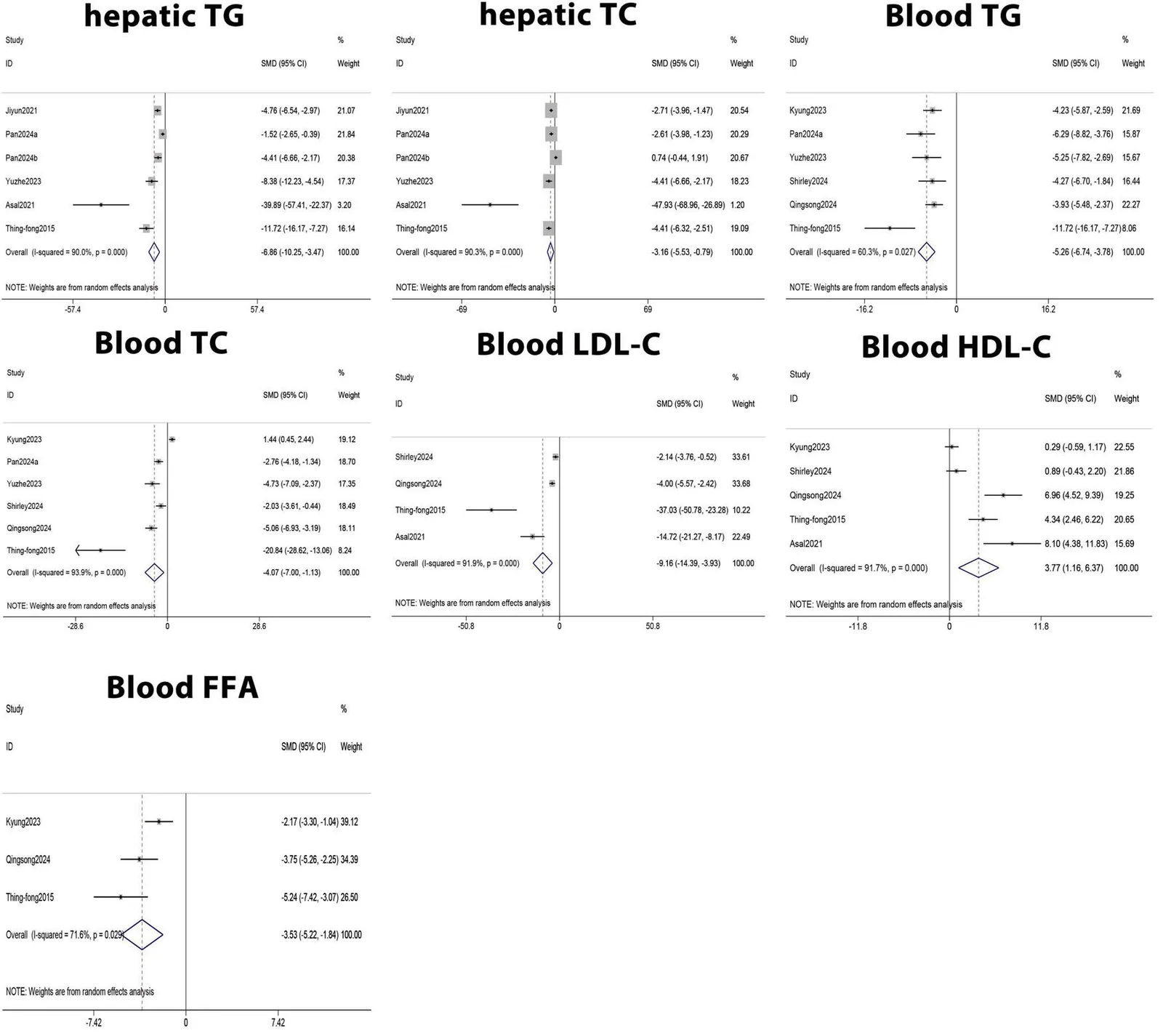
Forest plots of the effects of 6-gingerol on hepatic and circulating lipid profiles in experimental NAFLD animal models. Effect sizes are presented as SMDs with 95% CIs using random-effects models. Heterogeneity among studies was assessed using the I^2^ statistic. Negative SMD values indicate reductions in lipid parameters, whereas positive SMD values indicate increases; the clinical or biological interpretation depends on the specific lipid outcome. NAFLD, non-alcoholic fatty liver disease; TG, triglycerides; TC, total cholesterol; LDL-C, low-density lipoprotein cholesterol; HDL-C, high-density lipoprotein cholesterol; FFA, free fatty acid; SMD, standardized mean difference; CI, confidence interval.

Pooled analyses indicated significant improvements in hepatic and circulating lipid parameters following 6-gingerol administration (Figure 3). Hepatic triglycerides were significantly reduced (6 studies; SMD −6.86, 95% CI −10.25 to −3.47; *P* < 0.001; *I*^2^ = 90.0%, *P* < 0.001), as were hepatic total cholesterol levels (6 studies; SMD −3.16, 95% CI −5.53 to −0.79; *P* = 0.009; *I*^2^ = 90.3%, *P* < 0.001). Similar benefits were observed in circulating lipid profiles, with reductions in serum triglycerides (6 studies; SMD −5.26, 95% CI −6.74 to −3.78; *P* < 0.001; *I*^2^ = 60.3%, *P* = 0.027), total cholesterol (6 studies; SMD −4.07, 95% CI −7.00 to −1.13; *P* = 0.007, *I*^2^ = 93.9%, *P* < 0.001), low−density lipoprotein cholesterol (LDL-C) (4 studies; SMD −9.16, 95% CI −14.39 to −3.93; *P* = 0.001; *I*^2^ = 91.9%, *P* < 0.001), and free fatty acids (FFA) (3 studies; SMD −3.53, 95% CI −5.22 to −1.84; *P* < 0.001; *I*^2^ = 71.6%, *P* = 0.029). In contrast, high-density lipoprotein cholesterol (HDL-C) levels were increased (5 studies; SMD 3.77, 95% CI 1.16−-6.37; *P* = 0.005; *I*^2^ = 91.7%, *P* < 0.001). Substantial heterogeneity was observed across all hepatic and circulating lipid outcomes. Nevertheless, the overall direction of the pooled effects remained favorable across these lipid parameters.

### 3.7 Liver function tests

**FIGURE 4:**
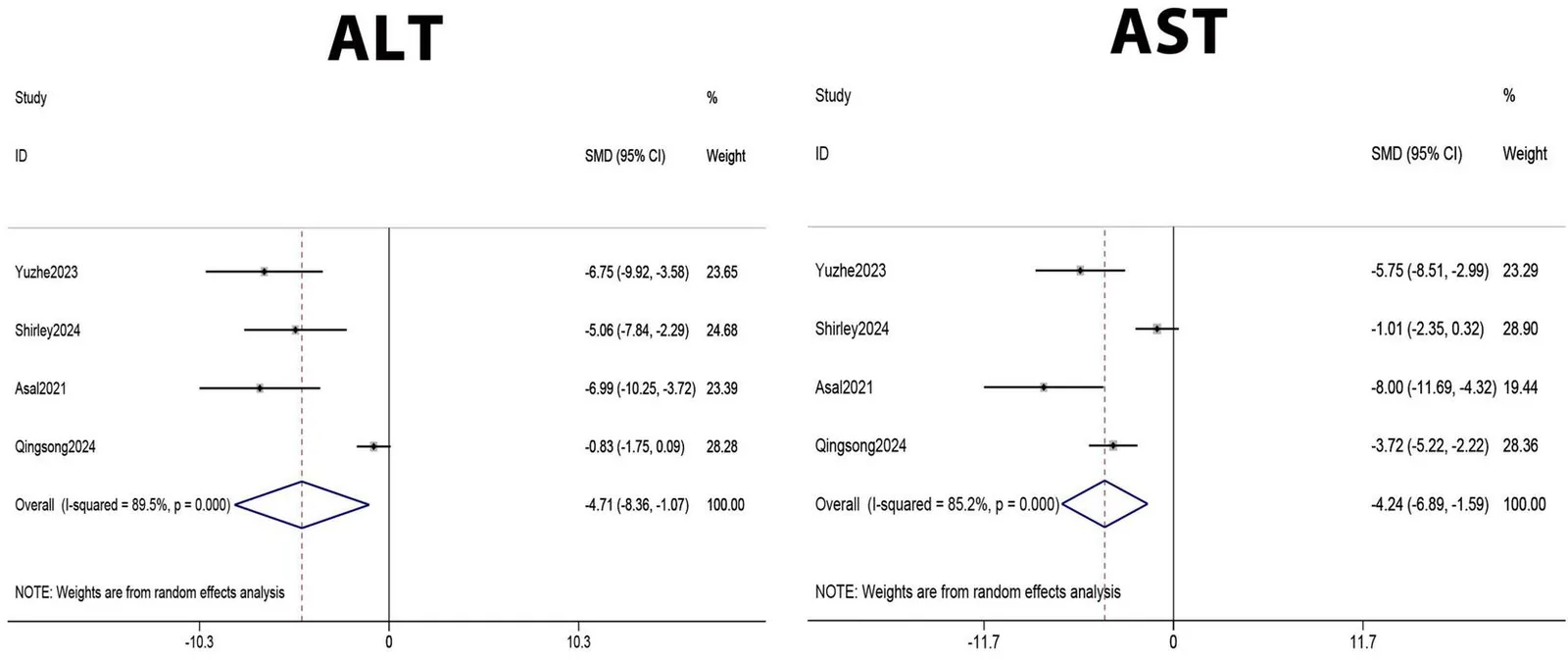
Forest plots showing the effects of 6-gingerol on liver injury markers in experimental NAFLD animal models. Effect sizes are presented as SMDs with 95% CIs using random-effects models. Heterogeneity among studies was assessed using the I^2^ statistic. Negative SMD values indicate reductions in serum liver injury markers following 6-gingerol treatment. NAFLD, non-alcoholic fatty liver disease; ALT, alanine aminotransferase; AST, aspartate aminotransferase; SMD, standardized mean difference; CI, confidence interval.

6-Gingerol treatment was associated with improved biochemical markers of liver injury (Figure 4). ALT levels were lower in treated animals compared with controls (4 studies; SMD −4.71, 95% CI −8.36 to −1.07; *P* = 0.011), with substantial heterogeneity (*I*^2^ = 89.5%, *P* < 0.001). AST was similarly reduced (4 studies; SMD −4.24, 95% CI −6.89 to −1.59; *P* = 0.002; *I*^2^ = 85.2%, *P* < 0.001).

### 3.8 Glucose metabolism and insulin resistance

**FIGURE 5:**
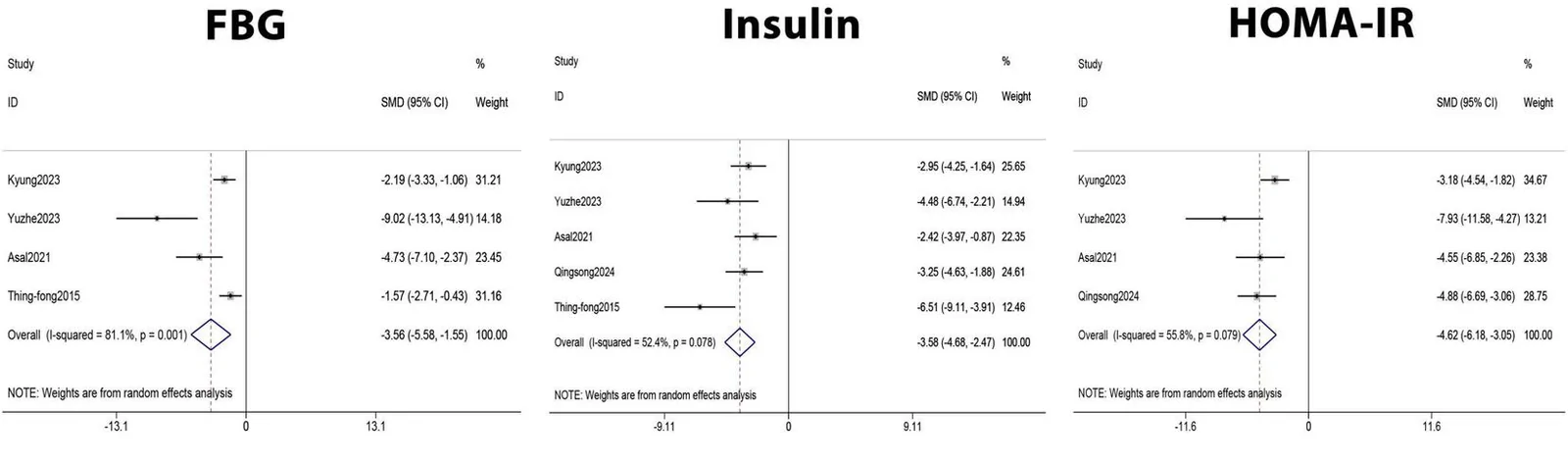
Forest plots of the effects of 6-gingerol on glucose metabolism and insulin resistance parameters in experimental NAFLD animal models. Effect sizes are presented as SMDs with 95% CIs using random-effects models. Heterogeneity among studies was assessed using the I^2^ statistic. Negative SMD values indicate improvements in glucose metabolism and insulin resistance following 6-gingerol treatment. NAFLD, non-alcoholic fatty liver disease; FBG, fasting blood glucose; HOMA-IR, homeostatic model assessment of insulin resistance; SMD, standardized mean difference; CI, confidence interval.

6-gingerol administration improved indices of glucose homeostasis and insulin resistance (Figure 5). FBG was reduced (4 studies; SMD −3.56, 95% CI −5.58 to −1.55; *P* = 0.001), with substantial heterogeneity (*I*^2^ = 81.1%, *P* = 0.001). Circulating insulin levels were also lower (5 studies; SMD −3.58, 95% CI −4.68 to −2.47; *P* < 0.001), with moderate heterogeneity (*I*^2^ = 52.4%, *P* = 0.078). HOMA-IR was significantly improved (4 studies; SMD −4.62, 95% CI −6.18 to −3.05; *P* < 0.001), with moderate heterogeneity (*I*^2^ = 55.8%, *P* = 0.079). Together, these results support favorable effects of 6-gingerol on systemic glucose regulation and insulin sensitivity.

### 3.9 Inflammation and metabolic regulatory molecules

**FIGURE 6:**
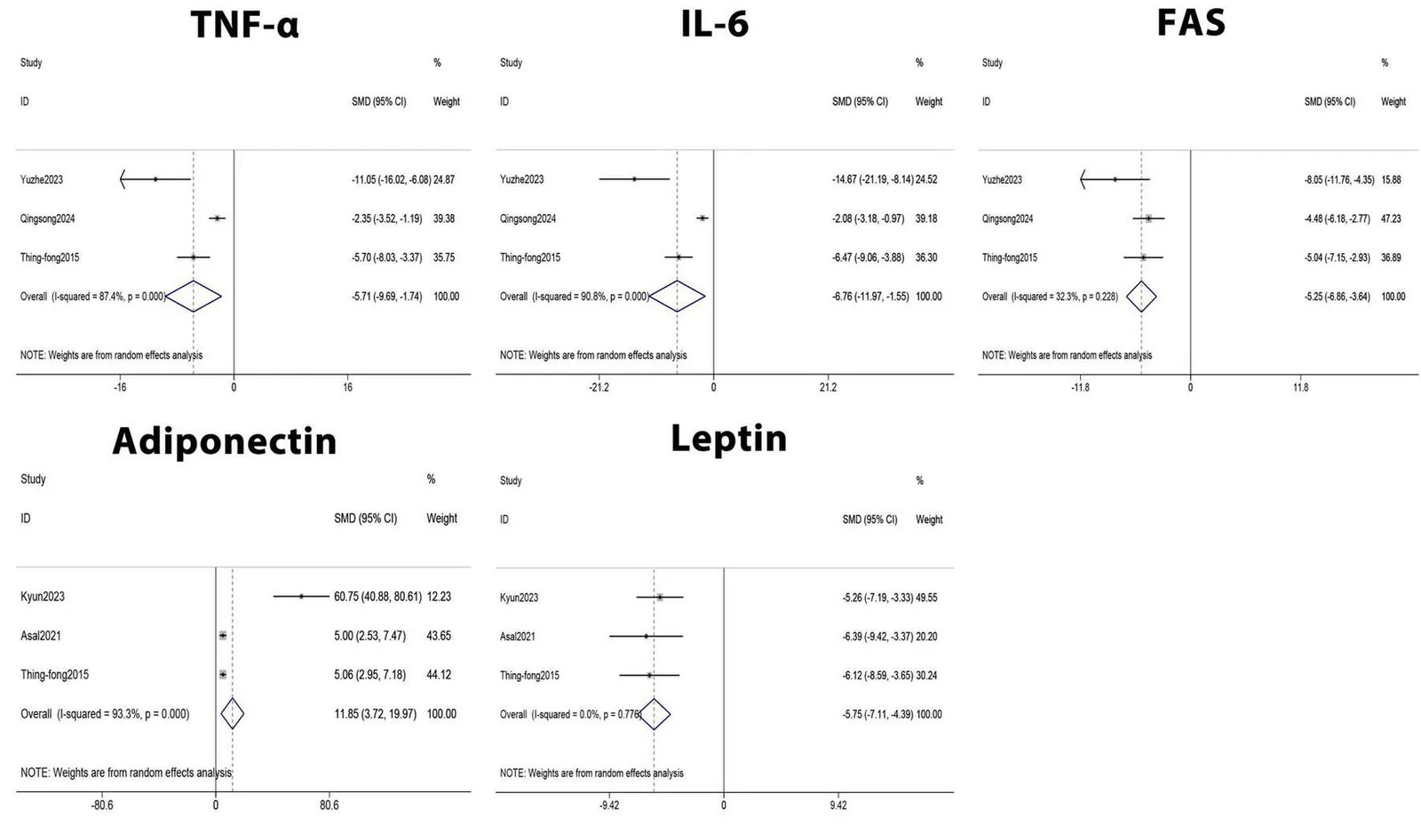
Forest plots showing the effects of 6-gingerol on inflammatory cytokines and metabolic regulatory molecules in experimental NAFLD animal models. Effect sizes are presented as SMDs with 95% CIs using random-effects models. Heterogeneity among studies was assessed using the I^2^ statistic. The direction of the favorable effect varies by outcome: lower TNF-α, IL-6, FAS, and leptin levels and higher adiponectin levels indicate beneficial modulation. NAFLD, non-alcoholic fatty liver disease; TNF-α, tumor necrosis factor-α; IL-6, interleukin-6; FAS, fatty acid synthase; SMD, standardized mean difference; CI, confidence interval.

Mechanistic meta-analyses suggested that 6-gingerol modulated inflammatory cytokines, lipogenesis-related signaling, and adipokines (Figure 6). Tumor necrosis factor-α (TNF-α) was reduced (3 studies; SMD −5.71, 95% CI −9.69 to −1.74; *P* = 0.005; *I*^2^ = 87.4%, *P* < 0.001), and interleukin-6 (IL-6) was similarly decreased (3 studies; SMD −6.76, 95% CI −11.97 to −1.55; *P* = 0.011; *I*^2^ = 90.8%, *P* < 0.001). 6-Gingerol reduced fatty acid synthase (FAS) expression (3 studies; SMD −5.25, 95% CI −6.86 to −3.64; *P* < 0.001), with low heterogeneity (*I*^2^ = 32.3%, *P* = 0.228). Adiponectin was increased (3 studies; SMD 11.85, 95% CI 3.72–19.97; *P* = 0.004) with substantial heterogeneity (*I*^2^ = 93.3%, *P* < 0.001), whereas leptin was reduced (3 studies; SMD −5.75, 95% CI −7.11 to −4.39; *P* < 0.001) with no detectable heterogeneity (*I*^2^ = 0.0%, *P* = 0.776). Overall, these findings indicate coordinated improvements in inflammatory and lipogenic signals and adipokine profiles, although effect magnitudes varied.

### 3.10 Subgroup analyses

Given the substantial heterogeneity observed across most outcomes, prespecified subgroup analyses were conducted to explore potential sources of between-study variability. Detailed results of the dose-, treatment duration-, and species-based subgroup analyses are presented in Tables 3–5.

**TABLE 3:** Dose-based subgroup analysis of the effects of 6-gingerol in NAFLD models.

Category	Outcome	Dose	Number of experiments	Heterogeneity (*P*-value)	SMD	95%CI
Anthropometric	BW	Low	3	93.1% ( < 0.001)	−2.61	−5.82,0.60
		High	5	93.2% ( < 0.001)	−3.05	−6.75,0.66
	Liver weight	Low	1	NA	−2.20	−3.34, −1.07
		High	4	85.4% ( < 0.001)	−6.77	−10.56, −2.98
	Liver index	Low	2	24.3% (0.250)	−4.19	−5.87, −2.51
		High	5	68.4% (0.013)	−3.84	−5.39, −2.29
Lipid profile	Hepatic TG	Low	3	82.7% (0.003)	−3.45	−5.78, −1.11
		High	3	83.7% (0.002)	−14.85	−23.56, −6.13
	Hepatic TC	Low	3	89.9% ( < 0.001)	−1.51	−3.81,0.79
		High	3	87.8% ( < 0.001)	−7.07	−12.62, −2.51
	Blood TG	Low	2	44.4% (0.180)	−5.03	−6.99, −3.06
		High	4	72.2% (0.013)	−5.62	−8.01, −3.22
	Blood HDL−C	Low	1	−-	0.29	−0.59, 1.17
		High	4	89.7% ( < 0.001)	4.83	1.54, 8.13
Glucose metabolism	FBG	Low	1	−-	−2.19	−3.33, −1.06
		High	3	87.2% ( < 0.001)	−4.67	−8.43, −0.91
	Insulin	Low	1	−-	−2.95	−4.25, −1.64
		High	4	61.8% (0.049)	−3.88	−5.39, −2.37
	HOMA-IR	Low	1	–	−3.18	−4.54, −1.82
		High	3	22.1% (0.277)	−5.26	−6.82, −3.70
Signaling protein	Adiponectin	Low	1	−-	60.75	40.88, 80.61
		High	2	0.0% (0.970)	5.04	3.43, 6.64

NAFLD, non-alcoholic fatty liver disease; SMD, standardized mean difference; 95%CI, 95% confidence interval; BW, body weight; TG, triglycerides; TC, total cholesterol; HDL-C, high-density lipoprotein cholesterol; FBG, fasting blood glucose; HOMA-IR, homeostatic model assessment for insulin resistance.

**TABLE 4:** Duration-based subgroup analysis of the effects of 6-gingerol in NAFLD models.

Category	Outcome	Duration	Number of experiments	Heterogeneity (*P*-value)	SMD	95%CI
Anthropometric	BW	Long	3	78.1% (0.010)	−4.40	−6.85, −1.96
		Short	5	93.8% ( < 0.001)	−1.93	−5.05,1.19
	Liver weight	Long	2	0.0% (0.654)	−2.34	−3.30, −1.38
		Short	3	67.98% (0.045)	−8.27	−11.74, −4.79
	Liver index	Long	5	56.1% (0.059)	−4.48	−5.94, −3.03
		Short	2	37.0% (0.208)	−2.64	−4.15, −1.13
Lipid profile	Hepatic TG	Long	2	87.7% (0.004)	-7.93	−14.73, −1.13
		Short	3	90.7% ( < 0.001)	−6.76	−11.59, −1.93
	Hepatic TC	Long	2	53.4% (0.143)	−3.40	−5.04, −1.76
		Short	4	92.7% ( < 0.001)	−3.45	−7.33,0.42
	Blood TG	Long	3	79.8% (0.007)	−6.10	−9.47, −2.73
		Short	3	24.5% (0.266)	−4.85	−6.26, −3.43
	Blood LDL−C	Long	2	95.9% ( < 0.001)	−18.89	−53.05,15.27
		Short	1	89.7% (0.002)	−8.87	−19.33, −1.59
	Blood HDL-C	Long	3	86.3% (0.001)	1.69	−0.36,3.73
		Short	2	0.0% (0.614)	7.30	5.26,9.34
Glucose metabolism	FBG	Long	2	0.0% (0.448)	−1.88	−2.69, −1.08
		Short	2	68.2% (0.076)	−6.53	−10.68, −2.30
	Insulin	Long	2	82.6% (0.016)	−4.54	−8.01, −1.07
		Short	3	9.2% (0.332)	−3.17	−4.16, −2.18
	HOMA-IR	Long	1	−	−3.18	−4.54, −1.82
		Short	3	22.1% (0.277)	−5.26	−6.82, −3.70
Signaling protein	TNF-α	Long	2	91.0% (0.001)	−6.35	−14.85, 2.14
		Short	1	−	−5.70	−8.03, −3.37
	IL-6	Long	2	92.8% ( < 0.001)	−7.94	−20.25, 4.37
		Short	1	−	−6.47	−9.06, −3.88
	Adiponectin	Long	2	96.6% ( < 0.001)	31.99	−22.55, 86.53
		Short	1	–	5.00	2.53, 7.47

NAFLD, non-alcoholic fatty liver disease; SMD, standardized mean difference; 95%CI, 95% confidence interval; BW, body weight; TG, triglycerides; TC, total cholesterol; LDL-C, low-density lipoprotein cholesterol; HDL-C, high-density lipoprotein cholesterol; FBG, fasting blood glucose; HOMA-IR, homeostatic model assessment for insulin resistance; TNF, tumor necrosis factor; IL-6, interleukin-6.

**TABLE 5:** Species-based subgroup analysis of the effects of 6-gingerol in NAFLD models.

Category	Outcome	Species	Number of experiments	Heterogeneity (*P*-value)	SMD	95%CI
Anthropometric	BW	m	5	95.2% ( < 0.001)	−2.96	−7.21,1.28
		r	2	0.0% (0.677)	−2.05	−3.03, −1.07
		h	1	−	−4.42	−6.33, −2.51
	Liver weight	m	4	90.1% ( < 0.001)	−6.65	−10.84, −2.46
		r	1	–	−2.69	−4.50, −0.88
	Liver index	m	4	65.7% (0.033)	−5.00	−7.05, −2.95
		r	2	0.0% (0.917)	−3.63	−4.95, −2.31
		h	1	–	−2.09	−3.34, −0.84
Lipid profile	Hepatic TG	m	4	83.7% ( < 0.001)	-7.31	−11.40, −3.23
		r	1	–	−1.52	−2.65, −0.39
		h	1	–	−11.72	−16.17, −7.27
	Hepatic TC	m	3	93.0% ( < 0.001)	−3.43	−7.23,0.36
		r	1	–	−2.61	−3.98, −1.23
		h	1	–	−4.41	−6.32, −2.51
	Blood TG	m	3	0.0% (0.687)	-4.26	−5.29, −3.23
		r	2	21.7% (0.259)	−5.25	−7.23, −3.27
		h	1	–	−11.72	−16.17, −7.27
	Blood HDL-C	m	3	94.8% ( < 0.001)	4.94	−0.64,10.52
		r	1	–	0.89	−0.43,2.20
		h	1	–	4.34	2.46,6.22
Glucose metabolism	FBG	m	3	83.75 (0.002)	−4.82	−8.15, −1.48
		h	1	–	−1.57	−2.71, −0.43
	Insulin	m	4	0.0% (0.518)	-3.08	−3.85, −2.32
		h	1	–	−6.51	−9.11, −3.91
Signaling protein	TNF-α	m	2	91.0% (0.001)	−6.35	−14.85,2.14
		h	1	–	−5.70	−8.03, −3.37
	IL-6	m	2	92.8% ( < 0.001)	−7.94	−20.25,4.37
		h	1	–	−6.47	−9.06, −3.88
	Adiponectin	m	2	96.6% ( < 0.001)	31.97	-22.63,86.57
		h	1	−	5.06	2.95,7.18

NAFLD, non-alcoholic fatty liver disease; SMD, standardized mean difference; 95%CI, 95% confidence interval; m, mouse; r, rat; h, hamster; BW, body weight; TG, triglycerides; TC, total cholesterol; HDL-C, high-density lipoprotein cholesterol; FBG, fasting blood glucose; TNF, tumor necrosis factor; IL-6, interleukin-6.

For dose-based subgroup analyses, studies were classified as high-dose ( ≥ 100 mg/kg/day) or low-dose ( < 100 mg/kg/day). Stratification by dose was associated with lower I^2^ values for several outcomes (Table 3). In particular, an I^2^ value of 0% was observed for adiponectin, and lower I^2^ values were observed for liver index, blood TG, and HOMA-IR. However, these descriptive changes should be interpreted cautiously because of the small number of studies within several strata. For several other outcomes, including liver weight, hepatic triglycerides, hepatic total cholesterol, and HDL-C, heterogeneity remained substantial. These findings suggest that dose may contribute to variability in treatment effects but does not fully account for the observed heterogeneity.

For treatment duration, studies were categorized as long-duration (≥8 weeks) or short-duration (<8 weeks). Stratification by treatment duration was associated with lower I^2^ values across a broader range of outcomes than the other stratifications (Table 4). I^2^ values of 0% were observed in some treatment-duration strata for liver weight, hepatic triglycerides, HDL-C, and fasting blood glucose. However, these values should not be interpreted as evidence of true homogeneity because the corresponding strata contained very few studies. Additional outcomes, such as blood triglycerides, hepatic total cholesterol, and insulin, also showed substantial reductions in heterogeneity. These descriptive findings suggest that treatment duration may contribute to between-study variability.

Subgroup analyses by species were also associated with lower I^2^ values for a subset of outcomes. Stratification by species was associated with I^2^ values of 0% for several endpoints, including body weight, liver index, hepatic total cholesterol, blood triglycerides, and insulin. Nevertheless, these findings were based on small and unbalanced subgroup strata and should be interpreted cautiously. By contrast, heterogeneity remained relatively high for other outcomes, such as hepatic triglycerides and FBG (Table 5). These descriptive findings suggest that species differences may contribute to heterogeneity, although their interpretation is limited by the small and unbalanced numbers of studies across subgroups.

Collectively, dose, treatment duration, and species may contribute to the observed between-study heterogeneity. Treatment-duration stratification was descriptively associated with lower I^2^ values across a greater number of outcomes than dose- or species-based stratification. However, several subgroup strata included only one or a few studies and were small and unbalanced. Consequently, reductions in I^2^, including values of 0%, should not be interpreted as evidence of true homogeneity or definitive effect modification. These subgroup analyses should therefore be regarded as exploratory and hypothesis-generating. Residual heterogeneity also remained for several outcomes, suggesting that additional factors, such as differences in NAFLD induction models, experimental design, and outcome assessment methods, may contribute to between-study variability.

### 3.11 Sensitivity analyses

Sensitivity analyses were performed for outcomes with sufficient numbers of studies, including body weight, liver weight, liver index, hepatic triglycerides, hepatic total cholesterol, blood triglycerides, blood total cholesterol, HDL-C, and insulin (Supplementary Figures 3–5). Leave-one-out analyses showed that the overall direction and statistical significance of the pooled effects remained unchanged across all outcomes, indicating that no single study had a disproportionate influence on the results. Although minor variations in effect size were observed in some cases, these did not materially affect the overall conclusions.

Publication bias was not assessed for any outcome, as fewer than 10 studies were available in each analysis. Funnel plots and statistical tests such as Egger’s or Begg’s tests are considered unreliable and underpowered in small-sample meta-analyses.

Replacing the highest-dose treatment arm with the lowest-dose treatment arm in studies with multiple intervention doses did not materially alter the pooled results. The overall direction of the pooled effects remained consistent across anthropometric indices, lipid metabolism, glucose metabolism, liver function, and inflammatory/metabolic markers (Supplementary Figures 6–10).

## 4 Discussion

### 4.1 Principal findings

In this systematic review and meta-analysis of controlled animal studies, we synthesized the preclinical evidence regarding the effects of 6-gingerol in experimental models of NAFLD. Overall, the findings suggest that 6-gingerol administration may be associated with improvements across multiple NAFLD-relevant domains, including anthropometric, metabolic, and hepatic outcomes. These effects were accompanied by favorable modulation of inflammatory mediators, lipogenic signaling molecules, and adipokines in a subset of included studies.

Overall, pooled analyses suggested favorable effects across the evaluated outcome domains. Despite differences in animal species, NAFLD induction methods, doses, and treatment durations, the overall direction of the observed effects appeared to be generally similar across the included studies. However, these findings should be interpreted with caution given the limited number of studies and the substantial between-study heterogeneity. At the same time, the observed heterogeneity underscores the influence of model- and design-related factors and highlights the need for greater standardization in future animal studies.

Improvements in hepatic lipid accumulation and liver enzymes were paralleled by favorable changes in systemic metabolic parameters, including glucose homeostasis and insulin resistance. This pattern suggests that the effects of 6-gingerol extend beyond local hepatic actions to involve systemic metabolic regulation. Mechanistic outcomes were not uniformly reported across studies and were available only in a subset of experiments, indicating that the present findings should be interpreted as associations with mechanistic signals rather than definitive pathway-level evidence.

This meta-analysis summarizes the available animal evidence regarding the effects of 6-gingerol in NAFLD models. Although favorable changes were observed across several outcome domains, the limited evidence base and inconsistent mechanistic reporting constrain both mechanistic and translational interpretation.

### 4.2 Mechanistic findings and contextual evidence

This section distinguishes mechanistic evidence reported in the included studies from contextual evidence derived from the broader experimental literature. The former includes both quantitatively pooled outcomes and findings summarized narratively, whereas the latter is used to interpret biological plausibility and generate hypotheses. Evidence from studies outside the eligibility set was not included in the quantitative synthesis and should not be regarded as a mechanism established by the present meta-analysis.

#### 4.2.1 Mechanistic evidence from the included NAFLD studies

Mechanistic endpoints were reported in only a subset of the included studies and were less consistent than the main metabolic outcomes. FAS, TNF-α, IL-6, adiponectin, and leptin were available from at least three independent studies and could be quantitatively synthesized. Oxidative stress-related outcomes were more heterogeneous and were examined narratively.

The pooled analysis showed lower FAS expression after 6-gingerol treatment. This result was based on the studies by Xia et al. (15), Liu et al. (20), and Tzeng et al. (22). Lower FAS expression was observed in parallel with reductions in hepatic triglyceride and total cholesterol levels. The consistency in direction between these outcomes suggests that suppression of hepatic lipid synthesis may have contributed to the improvement in steatosis. The available studies, however, did not assess the same upstream signaling molecules, and no common pathway could be identified from the pooled data. TNF-α and IL-6 were also reduced following 6-gingerol administration (15, 20, 22). Both analyses were based on three studies, and substantial heterogeneity remained. The findings support a reduction in inflammatory activity, but the magnitude of this response varied across experimental settings. Because the included studies did not consistently measure pathway-level inflammatory markers, these cytokine results cannot be used to attribute the effect to a specific signaling cascade.

The adipokine findings came from a partly different set of experiments. Adiponectin increased, whereas leptin decreased, in the studies by Sarrafan et al. (12), Hong et al. (14), and Tzeng et al. (22). These changes occurred alongside lower insulin concentrations and improved HOMA-IR in the broader metabolic analyses. They are therefore compatible with an improvement in systemic metabolic regulation rather than an exclusively hepatic response. Whether the changes in adiponectin and leptin directly contributed to improved insulin sensitivity was not examined, and mediation cannot be inferred from the current data.

**FIGURE 7:**
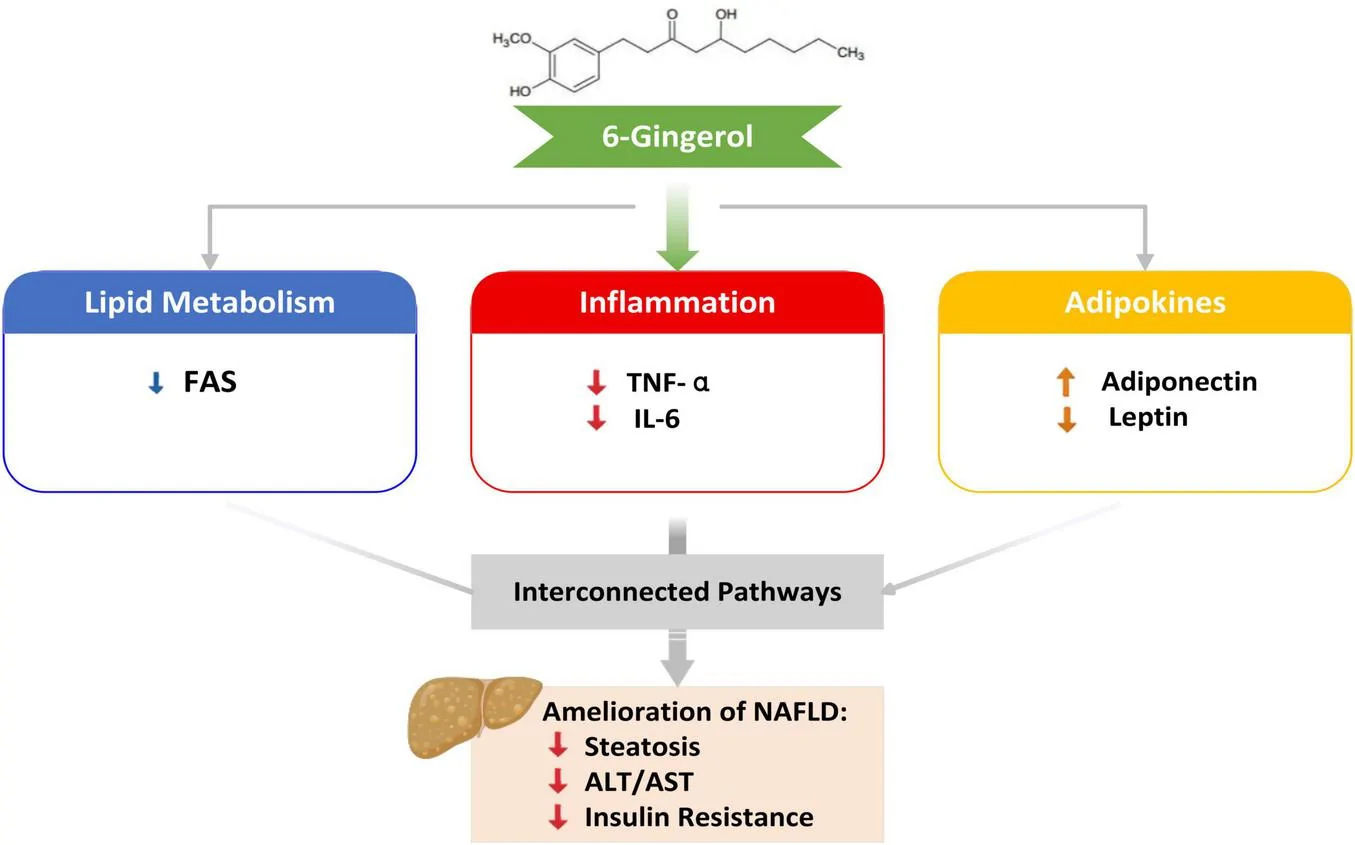
Schematic summary of the mechanistic and metabolic findings reported in the studies included in the present review. The figure depicts quantitatively pooled changes in fatty acid synthase (FAS), tumor necrosis factor-α (TNF-α), interleukin-6 (IL-6), adiponectin, leptin, liver injury markers, hepatic lipid-related outcomes, and insulin resistance following 6-gingerol administration in experimental NAFLD models. The connecting arrows represent an integrative conceptual framework and should not be interpreted as demonstrating causal pathways. NAFLD, non-alcoholic fatty liver disease; FAS, fatty acid synthase; TNF-α, tumor necrosis factor-α; IL-6, interleukin-6; ALT, alanine aminotransferase; AST, aspartate aminotransferase.

Oxidative stress could not be evaluated in the same way. The biomarkers differed among studies, and no individual oxidative stress marker was reported by three independent experiments. Liu et al. nevertheless reported improvements in oxidative stress-related measures following 6-gingerol treatment (20). This observation provides supporting evidence, but it remains an individual-study finding rather than a pooled estimate. Figure 7 presents the mechanistic and metabolic signals that were reported in the included studies. The connections shown in the figure are intended to organize these findings visually and do not indicate that causal relationships between the individual outcomes were demonstrated.

#### 4.2.2 Contextual evidence from the broader experimental literature

Evidence from studies outside the eligibility set provides additional biological context for interpreting the mechanistic signals identified in the included NAFLD studies. These data were not part of the quantitative synthesis and do not establish that the same pathways mediated the pooled effects. In an age-related hepatic steatosis model, 6-gingerol was reported to regulate lipogenesis and fatty-acid oxidation while also improving mitochondrial function and oxidative stress (5). These findings provide a plausible biological context for the lower FAS expression and hepatic lipid levels observed in the present meta-analysis. The experimental settings were different, however, and the available evidence does not show that a common upstream pathway operated across the included NAFLD models.

Oxidative and inflammatory regulation may also overlap. He et al. reported that 6-gingerol activated Nrf2 and reduced inflammatory injury in hyperlipidemic mice (2). Nrf2 regulates cellular antioxidant responses through downstream effectors such as HO-1 and NQO1 and is functionally linked to broader antioxidant enzyme systems involving SOD, CAT, and GPx (23). These defenses may reduce oxidative injury and limit the accumulation of mitochondrial ROS. This framework is consistent with the oxidative stress-related observations reported by individual included studies, as well as the pooled reductions in TNF-α and IL-6. It remains a contextual interpretation: Nrf2 activation was not evaluated consistently enough in the eligible studies to be synthesized, and the cytokine findings alone cannot identify the pathway through which inflammation was reduced. Beyond hepatic redox and inflammatory responses, external studies also suggest that 6-gingerol may affect adipose tissue. In high-fat diet-induced obese mice, Cheng et al. found improvements in adipocyte hypertrophy and hyperplasia after treatment (24). Gunawan et al. subsequently reported changes in adipocytokines and insulin resistance in a rat model of metabolic syndrome (25). These studies make altered adipose tissue function a reasonable explanation for the pooled increase in adiponectin and decrease in leptin. They do not demonstrate adipose–liver mediation in the NAFLD experiments included here.

Fibrosis-related endpoints, including TGF-β, α-SMA, and collagen deposition, were not systematically assessed in the included NAFLD studies and were therefore not eligible for quantitative synthesis. Possible antifibrotic effects of 6-gingerol remain uncertain. In cardiac models, 6-gingerol has been reported to limit pathological remodeling and profibrotic signaling (26). Reduced collagen deposition has also been observed in a pulmonary fibrosis model (27). Both findings come from organs and disease settings outside the scope of this review. They provide a rationale for examining TGF-β signaling, α-SMA expression, and extracellular matrix deposition in future NAFLD studies, but should not be interpreted as evidence that 6-gingerol prevented or reversed hepatic fibrosis in the included experiments.

The broader literature on mTOR may also help contextualize the LKB1/AMPK signaling reported by Liu et al. in one of the included studies (20). mTOR is closely involved in metabolic responses to changes in cellular energy and nutrient supply (28, 29), and its transcriptional functions may contribute to metabolic remodeling under conditions of nutrient excess (30). Such interactions provide a possible framework for interpreting the concurrent changes in hepatic lipids, glucose regulation, inflammatory markers, and adipokines across the included studies. Direct evidence linking 6-gingerol to the complete LKB1-AMPK-mTOR axis, particularly its mTOR component, remains limited; this framework should therefore be regarded as hypothesis-generating rather than as a conclusion of the present meta-analysis.

### 4.3 Sources of heterogeneity and subgroup findings

Substantial heterogeneity was observed across many pooled outcomes in this meta-analysis, which is a common feature of preclinical animal research (11). Such heterogeneity likely reflects methodological and biological variability inherent to animal studies, including differences in species and strains, NAFLD induction methods, dosing regimens, treatment duration, and outcome assessment protocols (11). These factors can markedly influence both baseline disease severity and responsiveness to intervention, thereby contributing to variability in effect size estimates.

To explore potential sources of heterogeneity, prespecified subgroup analyses were conducted according to animal species, 6-gingerol dose, and treatment duration. Although the number of studies within individual subgroups was limited for some outcomes, treatment effects generally showed similar directional trends across subgroup categories. Stratification was associated with lower I^2^ values for several outcomes, including I^2^ values of 0% in some strata. However, because several strata contained only one or a few studies, these descriptive changes should not be interpreted as evidence of true homogeneity or definitive effect modification.

Descriptively, some higher-dose and longer-duration strata showed larger effect estimates or lower I^2^ values. However, because several subgroup strata contained only one or a few studies and were small and unbalanced, these patterns cannot establish dose-response or duration-response relationships. Similarly, I^2^ values of 0% in small subgroup strata should not be interpreted as evidence of true homogeneity. No definitive conclusions regarding species, dose, or treatment duration as effect modifiers can be drawn from the current evidence. These subgroup findings should therefore be considered exploratory and hypothesis-generating.

Despite heterogeneity in effect size, the overall pattern of effects was generally similar across most outcomes. This pattern suggests that favorable effects were observed across diverse experimental settings despite quantitative differences in effect size. Nevertheless, the limited number of studies within certain subgroups restricts definitive conclusions regarding moderators of treatment response.

Future preclinical studies would benefit from greater standardization of NAFLD models, dosing strategies, and outcome measures, as well as transparent reporting of experimental design and methodological details. Such improvements would facilitate more precise estimation of treatment effects and enhance the interpretability and reproducibility of animal meta-analyses.

### 4.4 Implications for translational research and clinical relevance

NAFLD remains an area of substantial unmet clinical need, with limited approved pharmacological options and no universally effective therapy (8, 31, 32). In this context, bioactive natural compounds with multi-target metabolic effects and favorable safety profiles have attracted increasing attention as potential adjunctive or preventive strategies (8, 11). The present meta-analysis provides a quantitative synthesis of preclinical evidence suggesting that 6-gingerol may exert broad metabolic benefits in NAFLD animal models, supporting its continued investigation as a candidate for translational research.

The observed improvements across hepatic lipid accumulation, liver enzymes, glucose metabolism, and inflammatory and adipokine-related markers align with key pathophysiological features of NAFLD. Such multi-domain effects are particularly relevant given the complex and multifactorial nature of the disease, which limits the effectiveness of single-target interventions. From a translational perspective, these findings suggest that 6-gingerol may hold promise as part of a multi-target therapeutic approach, rather than as a stand-alone agent aimed at a single pathway.

However, several considerations must be addressed before extrapolating these findings to human disease. The doses of 6-gingerol used in animal studies often exceed typical dietary exposure, and the pharmacokinetics, bioavailability, and long-term safety of purified 6-gingerol in humans remain incompletely characterized. In addition, heterogeneity in animal models and experimental designs complicates direct translation of effect sizes to clinical settings. These factors highlight the need for carefully designed dose-finding, pharmacokinetic, and safety studies, followed by well-controlled clinical trials.

Importantly, the present synthesis helps define priorities for future research by identifying outcome domains and mechanistic signals that appear most responsive to 6-gingerol. Standardized preclinical experiments incorporating clinically relevant dosing regimens, rigorous methodological reporting, and harmonized outcome measures would substantially strengthen the translational evidence base. Ultimately, integration of high-quality preclinical data with early-phase clinical studies will be essential to determine whether the metabolic benefits observed in animal models can be meaningfully translated to patients with NAFLD.

### 4.5 Strengths and limitations

This study has several strengths. First, to our knowledge, this is the first systematic review and meta-analysis to quantitatively synthesize the effects of 6-gingerol in animal models of NAFLD. The review protocol was prospectively registered in PROSPERO, and the study was conducted and reported in accordance with PRISMA 2020, enhancing methodological transparency and reducing the risk of selective reporting. Second, we applied random-effects models throughout and used standardized mean differences to accommodate variability in outcome measurement, reporting scale, and baseline outcome ranges across the included studies. Third, a broad range of NAFLD-relevant outcome domains was evaluated, allowing an integrated assessment of anthropometric, metabolic, hepatic, and mechanistic signals. Finally, risk of bias was systematically assessed using SYRCLE’s tool, and prespecified subgroup analyses were conducted to explore potential sources of heterogeneity (33, 34).

Several limitations should also be acknowledged. First, the number of included studies for individual outcomes was relatively small, particularly for mechanistic endpoints, which limits the precision of pooled estimates and the strength of mechanistic inferences.

Second, substantial heterogeneity was observed for many outcomes, reflecting differences in animal species and strains, NAFLD induction methods, dosing regimens, routes of administration, treatment duration, and outcome assessment methods.

Although prespecified subgroup analyses were performed, several subgroup strata contained only one or a few studies and were small and unbalanced. Accordingly, these subgroup findings should be regarded as exploratory and hypothesis-generating rather than as definitive evidence of effect modification. Reductions in I^2^, including values of 0%, should also be interpreted cautiously when based on very few studies. In addition, the limited number of studies contributing to each outcome precluded reliable meta-regression analyses, which would have been underpowered and susceptible to unstable or spurious associations.

Variation in NAFLD induction models may also have contributed to the observed heterogeneity. The majority of included studies used high-fat diet models, which primarily reproduce obesity-associated metabolic dysfunction and insulin resistance. In contrast, fructose-containing models, including high-fructose and combined high-fat/high-fructose diets, promote hepatic de novo lipogenesis and may induce broader metabolic disturbances. These pathophysiological differences may have contributed to the between-study heterogeneity observed in the present meta-analysis. The route of administration may represent another potential source of variability across studies. Oral gavage generally provides more precise dose delivery, whereas dietary supplementation may result in greater variability in actual intake because of differences in food consumption among animals. Such heterogeneity is a well-recognized feature of preclinical animal meta-analyses, arising from inherent variability in experimental design and disease modeling. However, it may still compromise the robustness and generalizability of pooled estimates (11).

Third, methodological reporting was often incomplete, resulting in unclear risk of bias across several domains; such limitations may also have contributed to the observed heterogeneity and uncertainty in effect estimates.

Several pooled SMDs were unusually large. Although these estimates may partly reflect genuine biological effects of 6-gingerol in experimental NAFLD, several methodological and experimental factors may also have contributed. Because an SMD expresses the mean difference relative to the within-study standard deviation, highly homogeneous animal populations and tightly controlled experimental conditions may yield small within-group standard deviations and consequently large standardized effects. The small group sizes used in many preclinical experiments may further produce imprecise and unstable estimates of both group means and standard deviations. Differences in animal species and strains, NAFLD induction methods, baseline disease severity, dose, treatment duration, laboratory assays, and outcome-normalization procedures may also have contributed to variability in effect magnitude. In addition, small-study effects and selective publication of positive findings cannot be excluded, because the number of studies contributing to each outcome was insufficient for a reliable assessment of publication bias. SMDs are also less directly interpretable than absolute mean differences. Accordingly, greater emphasis should be placed on the direction and uncertainty of the pooled effects than on the absolute magnitude of the SMDs, and these estimates should not be interpreted as directly transferable clinical effect sizes.

Furthermore, when multiple doses of 6-gingerol were evaluated within a single study, only the highest-dose arm was included in the primary analysis according to the prespecified protocol. This approach avoided non-independence arising from a shared control group and provided a consistent arm-selection rule across studies; however, it may also have increased the magnitude of some pooled estimates. The additional lowest-dose sensitivity analysis yielded a similar overall direction of effect across the evaluated outcome domains, supporting the robustness of the directional findings. Nevertheless, some inflation in the magnitude of the primary pooled estimates due to selection of the highest-dose arm cannot be completely excluded.

Finally, all included studies were preclinical in nature, which limits the direct clinical translation of the present findings. A key translational limitation concerns the relevance of the animal doses to achievable human exposure. While favorable effects were observed across the included animal studies, the administered doses may not be readily achievable through habitual dietary ginger consumption. For example, using the body surface area conversion approach recommended by the U.S. Food and Drug Administration (FDA), a mouse dose of 100 mg/kg corresponds to approximately 8.1 mg/kg in humans, or about 570 mg/day for a 70-kg adult (35). However, such conversion provides only an approximate estimate and does not account for interspecies differences in absorption, metabolism, distribution, and elimination (35). This issue is particularly relevant because ginger phenolics, including 6-gingerol, undergo extensive phase II conjugation following oral administration, resulting in relatively low systemic exposure to the unconjugated parent compounds (36, 37). Therefore, future studies should incorporate pharmacokinetic assessment, formulation optimization, and clinically relevant dose selection before the preclinical effects observed here can be translated into human NAFLD studies.

Despite these limitations, favorable effects were observed across diverse experimental settings. These findings should be interpreted as evidence of potential efficacy in animal models rather than proof of clinical benefit. Further standardized preclinical experiments and well-controlled clinical studies are required to clarify the translational relevance of 6-gingerol in NAFLD. As additional preclinical evidence becomes available, the precision and reliability of the current estimates can be further strengthened.

## 5 Conclusion

This systematic review and meta-analysis demonstrates that 6-gingerol is associated with favorable effects across multiple outcome domains in animal models of non-alcoholic fatty liver disease, including anthropometric indices, hepatic and circulating lipid profiles, liver injury enzymes, glucose metabolism, and inflammatory and adipokine-related markers. These findings support the potential metabolic and hepatoprotective effects of 6-gingerol in experimental NAFLD models.

However, the available evidence is limited to animal studies and is characterized by substantial heterogeneity and variable methodological quality. Mechanistic outcomes were reported inconsistently and should be interpreted as supportive signals rather than definitive pathway-level evidence. Consequently, the present findings should not be directly extrapolated to clinical efficacy.

Future research should prioritize standardized and rigorously reported preclinical experiments, including harmonized NAFLD models, clinically relevant dosing strategies, and systematic assessment of mechanistic endpoints. Well-designed clinical studies will be required to determine the translational relevance, optimal dosing, and long-term safety of 6-gingerol in the management of NAFLD.

## Data Availability

The dataset used for the analyses in this study can be made available by the corresponding author upon reasonable request.
