# Supplementary material for: 6-gingerol modulates lipid metabolism, insulin resistance, and inflammation in NAFLD: evidence from a preclinical meta-analysis
Source: Front Nutr. 2026 Aug 13;13:1872764. doi: 10.3389/fnut.2026.1872764 (PMC13518360; doi:10.3389/fnut.2026.1872764)
Supplement: Supplementary file 1 [file Supplementary_file_1.docx]

Supplementary Material

# 1 Supplementary Tables

**Table S1 Complete Search Strategy for Web of Science Core Collection**

| #1 | TS=( Gingerol OR (6)-gingerol OR 6-gingerol) |
| --- | --- |
| #2 | TS=(Non-alcoholic Fatty Liver Disease OR Non alcoholic Fatty Liver Disease OR Fatty Liver, Nonalcoholic OR Fatty Livers, Nonalcoholic OR Liver, Nonalcoholic Fatty OR Livers, Nonalcoholic Fatty OR Nonalcoholic Fatty Liver OR Nonalcoholic Fatty Livers OR NAFLD OR Nonalcoholic Fatty Liver Disease OR Nonalcoholic Steatohepatitis OR Nonalcoholic Steatohepatitides OR Steatohepatitides, Nonalcoholic OR Steatohepatitis, Nonalcoholic OR MAFLD OR metabolic associated fatty liver disease OR MASLD OR metabolic dysfunction-associated steatotic liver disease OR metabolic dysfunction-associated fatty liver disease OR NAFLD OR NASH OR MASH OR metabolic associated steatohepatitis OR steatosis of liver OR steatohepatitis nonalcoholic OR metabolic associated steatohepatitis OR liver steatosis) |
| #3 | #1 AND #2 |

# Table S2 Complete Search strategy for the Cochrane Library

| #1 | [gingerol] explode all trees |
| --- | --- |
| #2 | (gingerol):ti,ab,kw OR ((6)-gingerol):ti,ab,kw OR (6-gingerol):ti,ab,kw |
| #3 | #1 OR #2 |
| #4 | [Non-alcoholic Fatty Liver Disease] explode all trees |
| #5 | (Non-alcoholic Fatty Liver Disease):ti,ab,kw OR (Non alcoholic Fatty Liver Disease):ti,ab,kw OR (Fatty Liver, Nonalcoholic):ti,ab,kw OR (Fatty Livers, Nonalcoholic):ti,ab,kw OR (Liver, Nonalcoholic Fatty):ti,ab,kw OR (Livers, Nonalcoholic Fatty):ti,ab,kw OR (Nonalcoholic Fatty Liver):ti,ab,kw OR (Nonalcoholic Fatty Livers):ti,ab,kw OR (NAFLD):ti,ab,kw OR (Nonalcoholic Fatty Liver Disease):ti,ab,kw OR (Nonalcoholic Steatohepatitis):ti,ab,kw OR (Nonalcoholic Steatohepatitides):ti,ab,kw OR (Steatohepatitides, Nonalcoholic):ti,ab,kw OR (Steatohepatitis, Nonalcoholic):ti,ab,kw OR (MAFLD):ti,ab,kw OR (metabolic associated fatty liver disease):ti,ab,kw OR (MASLD):ti,ab,kw OR (metabolic dysfunction-associated steatotic liver disease):ti,ab,kw OR (metabolic dysfunction-associated fatty liver disease):ti,ab,kw OR (NAFLD):ti,ab,kw OR (NASH):ti,ab,kw OR (MASH):ti,ab,kw OR (metabolic associated steatohepatitis):ti,ab,kw OR (steatosis of liver):ti,ab,kw OR (steatohepatitis nonalcoholic):ti,ab,kw OR (metabolic associated steatohepatitis):ti,ab,kw OR (liver steatosis):ti,ab,kw |
| #6 | #4 OR #5 |
| #7 | #3 AND #6 |

**Table S3 Complete Search strategy for EmBase**

| #1 | 'gingerol'/exp |
| --- | --- |
| #2 | 'gingerol':ti,ab,kw OR '(6)-gingerol':ti,ab,kw OR '6-gingerol':ti,ab,kw |
| #3 | #1 OR #2 |
| #4 | 'metabolic fatty liver'/exp OR 'nonalcoholic fatty liver'/exp |
| #5 | 'metabolic fatty liver':ti,ab,kw OR 'nonalcoholic fatty liver':ti,ab,kw OR 'dys-metabolism associated fatty liver disease':ti,ab,kw OR 'dysmetabolism associated fatty liver disease':ti,ab,kw OR 'dysmetabolism associated fld':ti,ab,kw OR mafld:ti,ab,kw OR 'metabolic associated fatty liver disease':ti,ab,kw OR 'metabolic associated fatty liver disease':ti,ab,kw OR 'metabolic dysfunction associated fatty liver disease':ti,ab,kw OR 'metabolic dysfunctionassociated fatty liver':ti,ab,kw OR 'metabolic fatty liver disease':ti,ab,kw OR 'metabolic fld':ti,ab,kw OR 'metabolism associated fatty liver disease':ti,ab,kw OR 'metabolic fatty liver':ti,ab,kw OR nafld:ti,ab,kw OR 'nonalcoholic fatty liver disease':ti,ab,kw OR 'non alcoholic fatty liver disease':ti,ab,kw OR 'non alcoholic hepato-steatosis':ti,ab,kw OR 'non alcoholic hepatosteatosis':ti,ab,kw OR 'non alcoholic liver steatosis':ti,ab,kw OR 'non alcoholic steatotic hepatopathy':ti,ab,kw OR 'non-alcoholic fatty liver':ti,ab,kw OR 'non-alcoholic fatty liver disease':ti,ab,kw OR 'non-alcoholic fld':ti,ab,kw OR 'non-alcoholic hepatic steatosis':ti,ab,kw OR 'nonalcoholic fatty liver disease':ti,ab,kw OR 'nonalcoholic fld':ti,ab,kw OR 'nonalcoholic hepatic steatosis':ti,ab,kw OR 'nonalcoholic hepatosteatosis':ti,ab,kw OR 'nonalcoholic liver steatosis':ti,ab,kw OR 'nonalcoholic fatty liver':ti,ab,kw |
| #6 | #4 OR #5 |
| #7 | #3 AND #6 |

# 2 Supplementary Figures

**Supplementary Figure S1.** Risk-of-bias graph showing the percentage of included studies judged as having low, unclear, or high risk of bias for each methodological domain.

**Supplementary Figure S2**. Risk-of-bias summary showing the review authors' judgments for each risk-of-bias domain across the individual included studies.

**Supplementary Figure S3.** Leave-one-out sensitivity analyses evaluating the robustness of the pooled estimates for anthropometric outcomes in experimental NAFLD animal models. Each analysis was performed by sequentially omitting one study at a time to assess the influence of individual studies on the overall pooled estimates. BW, body weight.

**Supplementary Figure S4.** Leave-one-out sensitivity analyses evaluating the robustness of the pooled estimates for hepatic and circulating lipid parameters in experimental NAFLD animal models. Each analysis was performed by sequentially omitting one study at a time to assess the influence of each individual study on the corresponding pooled estimates. TG, triglycerides; TC, total cholesterol; HDL, high-density lipoprotein cholesterol.

**Supplementary Figure S5.** Leave-one-out sensitivity analysis evaluating the robustness of the pooled effect estimate for blood insulin levels in experimental NAFLD animal models. Each analysis was conducted by sequentially omitting one study at a time to determine whether any individual study disproportionately influenced the overall result.

**Supplementary Figure S6. Sensitivity analysis using the lowest-dose treatment arm in studies with multiple intervention doses for anthropometric indices.** Effects of 6-gingerol on body weight, liver weight, and liver index in animal models of NAFLD. SMD, standardized mean difference; CI, confidence interval; BW, body weight; NAFLD, nonalcoholic fatty liver disease.

**Supplementary Figure S7. Sensitivity analysis using the lowest-dose treatment arm in studies with multiple intervention doses for lipid metabolism parameters.** Effects of 6-gingerol on hepatic TG, hepatic TC, blood TG, blood TC, blood LDL-C, blood HDL-C, and blood FFA in animal models of NAFLD. SMD, standardized mean difference; CI, confidence interval; TG, triglycerides; TC, total cholesterol; LDL-C, low-density lipoprotein cholesterol; HDL-C, high-density lipoprotein cholesterol; FFA, free fatty acids; NAFLD, non-alcoholic fatty liver disease.

**Supplementary Figure S8. Sensitivity analysis using the lowest-dose treatment arm in studies with multiple intervention doses for glucose metabolism parameters**. Effects of 6-gingerol on FBG, insulin, and HOMA-IR in animal models of NAFLD. SMD, standardized mean difference; CI, confidence interval; FBG, fasting blood glucose; HOMA-IR, homeostatic model assessment for insulin resistance; NAFLD, non-alcoholic fatty liver disease.

**Supplementary Figure S9. Sensitivity analysis using the lowest-dose treatment arm in studies with multiple intervention doses for liver function parameters.** Effects of 6-gingerol on ALT and AST in animal models of NAFLD. SMD, standardized mean difference; CI, confidence interval; ALT, alanine aminotransferase; AST, aspartate aminotransferase; NAFLD, non-alcoholic fatty liver disease.

**Supplementary Figure S10. Sensitivity analysis using the lowest-dose treatment arm in studies with multiple intervention doses for inflammatory markers and metabolism regulatory molecles.** Effects of 6-gingerol on TNF-α, IL-6, FAS, adiponectin, and leptin in animal models of NAFLD. SMD, standardized mean difference; CI, confidence interval; TNF-α, tumor necrosis factor-α; IL-6, interleukin-6; FAS, fatty acid synthase; NAFLD, non-alcoholic fatty liver disease..
